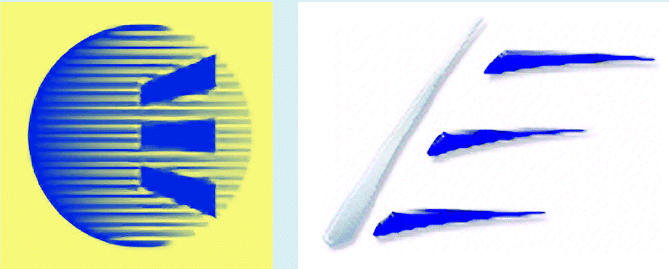# EHPnet: The Endocrine Society and the Society for Endocrinology

**Published:** 2005-10

**Authors:** Erin E. Dooley

The endocrine system encompasses the thyroid gland, the hypothalamus, the pancreas, the adrenal cortex, the thyroid, the parathyroid, and the male and female reproductive glands. Two large and well-established societies, The Endocrine Society and the Society for Endocrinology, serve the practitioners of this field. Both groups have established websites to keep their members and the general public aware of newsworthy events and developments in the field, and to educate those laypeople wishing to learn more about the subject.

The site of The Endocrine Society, http://www.endo-society.org/, features quick links so that information and materials can be pulled up by either subject or visitor’s role (e.g., clinician, student, volunteer worker). Among the 16 subjects featured are cardiovascular function, diabetes/insulin, genetics/genomics, male reproduction, and female reproduction. Also gathered in one area of the homepage are quick links to the society’s five publications, membership information, a member directory, and a subscription page.

The Endocrine Society’s homepage features two news sections, one of news in the general media, the other of updates within the society itself. Also available from the homepage is information on the society’s annual meeting, other society events, and related external events. Visitors can also select a quick link to The Hormone Foundation, the public education affiliate of the society, which offers basic information about the endocrine system, its function, and its associated diseases and disorders.

In the Press Room portion of the site is information on the America Weighs In campaign. This program focuses on educating the media, policy makers, and the general public about the role that endocrinologists play in researching and treating obesity. The Press Room also features a link to *The Endocrine Edge*, a free monthly online newsletter geared toward the public with the latest news from the society and the field of endocrinology.

The Society for Endocrinology site, http://www.endocrinology.org/, provides information about the Bristol, England–based organization and its programs. The society is affiliated with five journals, all accessible from a page on the website. The society also publishes a free quarterly newsletter, *The Endocrinologist*, which contains society news, general news, and feature articles.

The website also offers listings of grants and fellowships, society conferences, training courses sponsored by the society, and a calendar of events. The society sponsors a number of travel grants and has five research grant programs. There is also a page on books of interest to those in the field, which includes ordering information and short descriptions.

In 1997, the Society for Endocrinology established a committee for endocrinology nurses. This committee organizes conferences and an annual training course, and publishes the quarterly *Endocrine Nursing News* newsletter with reports from members and meeting notes. Past issues are available on the society website through the Endocrine Nurses link. Another subgroup are the Young Endocrinologists. Formed to support endocrinologists for up to six years after they receive their Ph.D., this group runs educational courses, provides career advice, and organizes special sessions at the Society of Endocrinology and British Endocrine Society meetings.

## Figures and Tables

**Figure:**